# “Knowledge I seek because culture doesn’t work anymore … It doesn’t work, death comes”: the experiences of third-generation female caregivers (*gogos*) in South Africa discussing sex, sexuality and HIV and AIDS with children in their care

**DOI:** 10.1186/s12889-021-10494-5

**Published:** 2021-03-09

**Authors:** Jane E. Simmonds, Charles D. H. Parry, Fareed Abdullah, Nadine Harker Burnhams, Nicola Christofides

**Affiliations:** 1 Office of AIDS and TB, South African Medical Research Council, Pretoria, South Africa; 2 School of Public Health, University of the Witwatersrand, Johannesburg, South Africa; 3 Alcohol, Tobacco and Other Drug Research Unit, South African Medical Research Council, Pretoria, South Africa; 4 Alcohol, Tobacco and Other Drug Research Unit, South African Medical Research Council, Cape Town, South Africa; 5 Department of Psychiatry, Stellenbosch University, Tygerberg, South Africa; 6 School of Public Health and Family Medicine, University of Cape Town, Cape Town, South Africa

**Keywords:** HIV, AIDS, Sexual communication, Grandmothers, Third-generation caregivers, South Africa, Sexual reproductive health, Sex

## Abstract

**Background:**

Sexual reproductive health communication between parents and children has been shown to promote safer sexual choices. In many South African households, third-generation female caregivers, often grandmothers or other older females, locally known as *gogos*, are primary caregivers of children due to parents being deceased or absent. Subsequently, the responsibility of talking about sex and related issues has shifted to these *gogos*. This study explored the experiences of *gogos* living in Alexandra, Johannesburg on talking about sex, sexuality and HIV and AIDS with children aged 10–18 years that are in their care.

**Methods:**

Ten primary caregivers were purposively selected. Data were collected through in-depth individual interviews. Thematic analysis was performed and inductive codes and themes identified.

**Results:**

All *gogos* selected found it difficult to discuss sex, sexuality and HIV and AIDS due to culture and traditional values impacting on personal experiences as well as generation and gender barriers. Perceived low self-efficacy due to low levels of knowledge and limited skills in speaking about sex, sexuality and HIV and AIDS also contributed to low levels of sexual reproductive health communication.

**Conclusions:**

This study highlights the need for interventions that focus on improving *gogos*’ knowledge about sexual reproductive health in addition to providing them with the skills to talk about sex, sexuality and HIV and AIDS with children in their care.

**Supplementary Information:**

The online version contains supplementary material available at 10.1186/s12889-021-10494-5.

## Background

Despite the declining HIV and AIDS prevalence globally, nearly half of all new HIV infections still occur among youth, especially in sub-Saharan Africa [1]. In South Africa, of the 231,000 new HIV infections in 2017, 38% were among persons aged 15–24 years, with around 66,000 of these new infections in girls and young women [2]. Adolescents are vulnerable to HIV infection as adolescence is a time of experimentation with sex [3] and when sexuality and sexual behaviour is shaped [4]. Adolescent sexual decision-making and behaviour are influenced by a number of factors at individual, peer, family, community and societal levels, with parents in particular playing a significant role [5]. Interventions that encourage the development of healthy sexual norms among adolescents can lead to a reduction in risk taking behaviour [4]. Research on the role of communication between parents and children in the development of adolescent sexual behaviour has established the importance of parent-child sexuality communication in reducing sexual risk behaviour [5–8].

Programmes to promote sexual communication with adolescents tend to focus on adolescents and/or mothers and sometimes both parents [5]. Globally, however, older people, usually older women and often grandmothers, are increasingly facing the challenges of raising children [9, 10]. In South Africa, many older women have become the primary caregivers of children orphaned by AIDS [11–13] and parents being absent from the home due to migration to other provinces in seek of work, job seeking, ill health or incarceration [10]. In 2018, it was estimated that nearly 4 million children in South Africa were living with a grandparent or an aunt [14]. There are a number of challenges facing these older caregivers which require new parenting strategies relevant to them, their experiences and their societies [10]. Among the challenges faced by these grandparents is the important issue of sexual and reproductive health (SRH) communication with the grandchildren in their care [15].

There is little available research globally on the role of grandmothers in SRH and the effectiveness of the grandparent-child dyad relationship in relation to SRH communication [16, 17]. In the United States, African-American grandparent caregivers felt unprepared to discuss topics regarding sexuality and sexual health with their grandchildren [18] and they required assistance communicating with their grandchildren about SRH because they belonged to a generation that found SRH topics embarrassing to discuss [19]. In spite of barriers to talking about sex, grandmothers wanted to have open and informed SRH conversations with their grandchildren [16, 17]. Parent-child conversations about SRH placed value on sexual abstinence as opposed to grandparent-child SRH conversations which addressed sex and sexuality [17].

In sub-Saharan Africa, interventions implemented in West and Southern Africa found that grandmothers can play an important role in SRH behaviour and choices of children in their care [20–22]. In a Ghanaian project, grandmothers served as important resources for reducing HIV infection and unintended pregnancy in adolescents. Both grandmothers and adolescents experienced the project’s intergenerational activities as beneficial [20]. In Malawi, trained “*agogos*” (the local name for grandmothers) helped young girls acquire sexual health information [21]. Both the trained “*agogos”*, and the girls they counselled, were more comfortable with SRH communication and were more likely to have interactive SRH discussions [21]. Meanwhile, in Botswana, grandmothers raising HIV-positive children found SRH conversations with these children very difficult due to cultural beliefs and that programmes to support these conversations between grandmothers and children were needed to address the needs of grandmothers in particular [22].

In South Africa, there is some research on the role of caregivers, who are not always biological parents, and how they communicate with children in their care about SRH issues [23, 24]. Grandparents in the Eastern Cape were unsuccessful in communicating SRH matters to the children they were raising and they needed to be supported in SRH communication with their grandchildren [25]. An evaluation of a family-centred adolescent HIV prevention programme, *Let’s Talk*, to address individual HIV transmission risk factors common among orphaned and vulnerable adolescents where nearly 50% of caregivers were grandmothers or aunts, found statistically significant improvements in adolescents’ HIV and condom use knowledge, as well as condom negotiation self-efficacy [26]. In addition, communication about healthy sexuality between caregiver and adolescent also improved [26].

Although there is limited research in South Africa on grandmothers and SRH communication with the children they are raising, there are studies that address the role of grandmothers in homes. Maternal grandmother involvement in adolescent adjustment was associated with more prosocial behaviour and less internalising of problems in adolescents in third-generation households [27]. Grandparent involvement in the emotional and behavioural health of adolescents was identified as a resource for promoting social and emotional competence in adolescents [28].

Literature on third-generation caregivers speaks to experiences related to age and frailty which are unique features of third-generation caring [13, 15]. Generational differences present their own specific challenges; for example child discipline and disharmony in the family in intergenerational relationships [13]. As a result, grandparents may not feel prepared to conduct SRH conversations, and this is exacerbated by the lack of resources to assist and prepare grandparents for SRH conversations [15, 22].

In this paper we document the experiences of older women, including grandmothers and aunts, referred to as *gogos*, in Alexandra, Johannesburg, of speaking to children aged 10 to 18 years in their care about sex, sexuality and HIV and AIDS. The findings are from interviews conducted as part of an evaluation of a skills-building workshop on SRH that aimed to improve *gogos* SRH communication skills. For the purposes of this paper, ‘grandmothers’ and ‘*gogos’* refer to “experienced, senior women in the household who are knowledgeable about all matters related to the health, development and well-being of children and their mothers. This includes not only maternal and paternal grandmothers, but also aunts and other older women” [29]. Although there was a range in ages of the older caregivers from 52 to 77 years, the women all self-identified as “*gogos*”. The terms “grandchild/grandchildren/children” relate to all children and adolescents being raised by a *gogo*, aged 10–18 years and therefore not necessarily just biological grandchildren.

The purpose of this paper is to describe and explore the *gogos*’ experiences and perceptions of talking about sex, sexuality and HIV and AIDS with the children they are raising.

## Methods

### Participants and study site

The qualitative study took place between February 2014 and January 2015. *Gogos* living in Alexandra, Johannesburg were recruited for the study by the researcher (JS) through a meeting held at a local non-profit organization (NPO). Alexandra is a crowded, low-income suburb with high levels of unemployment, poverty and crime. The women were selected because they were the primary caregivers of children between the ages of 10 and 18. Some *gogos* had other children who they were caring for outside of the specified age group where the household included children younger than 10 and older than 18 years but these dependents were not the focus of the SRH communication. All the women self-identified as *gogos* regardless of their age or whether they were raising biological grandchildren or not. They all attended weekly GOGO Days at Ratang Bana, a community-based NPO that cares for around 600 vulnerable children from the community. The GOGO Days were established primarily for attendees to work on income generation projects like crafting and vegetable growing.

We purposively selected ten *gogos* or older female caregivers who met the criteria of raising children aged 10–18. The reason for selecting this number of participants was to collect rich data at multiple timepoints including two rounds of in-depth interviews and focus group discussions. The *gogos* spoke and understood English as the intervention being evaluated was a training workshop conducted in English using a resource book, *HIV & AIDS* which was available in English [30]. The selected *gogos* were the primary caregivers of boys and/or girls within the specified age group. The women who were recruited were not all biological grandmothers of the children for whom they were caring.

### Data collection

Data were collected through in-depth individual interviews conducted in either Setswana or isiZulu by a trained research assistant. Pseudonyms were used in all transcripts and when reporting the findings. The research assistant was trained by the lead researcher (JES) on the purpose of the research study the data collection methods. The head of Ratang Bana in Alexandra granted permission to recruit participants for the study and informed consent was obtained from each *gogo* who expressed an interest in participating in the study. A separate consent form was used to get permission for creating audio-recordings of the interviews. The Human Research Ethics Committee at the University of the Witwatersrand approved the research study.

Interviews were held in either a room on the premises of Ratang Bana, which was private and designated for counselling at the centre, or at the homes of the individual participants depending on their preference. Basic socio-demographic information such as age, education, number of children in their care, age and sex of grand children being raised were collected, as well as sources of income including whether they were recipients of foster-care grants, old age “pensions” or other streams of income.

The interviews were guided by an interview guide with a scripted introduction, which was read at the beginning of each in-depth interview (See Additional File 1). The guide consisted of open-ended, non-directive questions, moving from the general to the specific. The interviewer started by asking about the *gogos*’ relationships with the children in their care in terms of activities, needs and demands, and whether there were problems experienced looking after the children. The interviewer then proceeded to questions specifically focusing on feelings about talking about sex, sexuality and HIV and AIDS; who should speak to children about these topics; what would make it easier to have these conversations; and what difficulties were experienced when talking about sex, sexuality and HIV and AIDS. The *gogos* were also asked about the last time they had talked to children in their care about sex, sexuality and HIV and AIDS, and if they had never spoken to their grandchildren about these topics the reasons why they had not. Interviews lasted for approximately 1 h.

### Data analysis

Audio recordings of the interviews were translated and transcribed verbatim. Identifying information was removed and pseudonyms used. Transcripts were imported into MAXQDA 10 for data coding and analysis. The thematic analysis was inductive, and codes and themes were developed after a close reading of the transcripts [31]. Text segments from the transcripts were assigned codes based on the ideas or categories being discussed. A code schema was developed with definitions for each code to facilitate further coding. The senior co-author (NC) reviewed the codes and definitions of each code and how they were applied to a transcript. Any ambiguity was discussed and clarified. Memos were written throughout the process of coding which tracked the patterns and co-occurring themes [31]. Mind maps were used to visualise the themes which were organised into patterns. These patterns were then reviewed and interpreted. Relevant salient quotes for each emergent theme were selected to support each theme during the process of writing up.

## Results

### Socio demographic characteristics of participants

Although all the older women in the sample self-identified as *gogos*, only two of the ten women were biological grandmothers and the others were aunts, great aunts, foster mothers, an adoptive mother and a caring older woman from the community. They cared for a combination of biological grandchildren, biologically-related children and other third-generation children. The average age of the women who participated in the study was 60.3 years, with a range from 52 to 77 years (Table 1). The average number of children being cared for by each *gogo* was three. There were 18 male and 18 female children being cared for by the *gogos* in the study. The average age of the child dependents in their care was 13.5 years with a range from 2 to 21 years, although the SRH communication focused on children between 10 and 18 years. None of the *gogos* were formally employed. Their sources of income were mainly social security grants and a few had some form of self-generated income. This self-generated income took the form of hawking, rentals and charitable support.

**Table 1:** Socio-demographic characteristics of *gogos*

Pseudonym	Age	Number of dependants	Ages of children in in *gogos* care	Source of income	Total monthly income disclosed in interview
Thoko	60–65	1	10–15	Senior Citizen grant Foster Care Grant	R2150
Thuli	60–65	3	15–20 (2), 20–25	Hawking Foster Care Grant	R840
Bontle	50–55	2	0–5, 15–20	Social Welfare Grant	R320
Sindi	55–60	4	0–5 (2), 5–10, 15–20	Rent Income Social Welfare Grant	R320
Florence	70–75	2	15–20, 20–25	Senior Citizen Grant Social Welfare Grant	R1670
Bongi	60–65	2	0–5, 15–20	Not disclosed	–
Lerato	50–60	2	10–15, 15–20	Social Welfare Grant Help from local NGO	R320
Agnes	50–60	3	10–15 (2) 15–20	Social Welfare Grant Monthly Stipend from Projects	R820
Mercy	50–55	2	10–15 (2)	Foster Care Grant	R840
Faith	70–89	2	10–15, 20–25	Foster Care Grant Senior Citizen Grant	R2190

R100 = approximately 6.5 Euros

### Experiences of the *gogos* talking about sex, sexuality and HIV and AIDS with their grandchildren

Various themes emerged regarding the *gogos*’ feelings and perceptions regarding talking about sex, sexuality and HIV and AIDS with their grandchildren. These included personal, contextual and structural barriers. On a personal level, barriers between *gogos* and grandchildren about talking about sex, sexuality and HIV and AIDS included the personal difficulties experienced by *gogos* due to past experiences with talking about sex, a lack of skills and knowledge of how to have these conversations, and a fear that talking about sex would encourage sexual behaviour. Contextual and structural barriers housed in cultural beliefs, gender and generational differences also affected the feelings and experiences of these *gogos* related to talking about sex, sexuality and HIV and AIDS.

### Personal barriers experienced by *gogos* in talking to grandchildren about sex

#### Difficulty talking about sex, sexuality and HIV and AIDS

All the participants expressed negative feelings regarding talking about sex, sexuality and HIV and AIDS and commented on the difficulty of speaking to their grandchildren about these issues:

“Hey lady …, that’s going to be difficult. That is going to be really difficult.” – *Sindi (55-59yrs, caregiver of four children)*

Although all the participants found talking about sex difficult, there was an acceptance or understanding amongst the participants that “how things are today is nothing like they used to be” (Bongi) and that there is now a need to talk to children about SRH. Lerato talked about trying to discuss issues that were not spoken about to her when she was growing up and how difficult this made talking about HIV and AIDS, and sex and sexuality.

Several *gogos* spoke about their lack of experience, knowledge and skills on this topic:

“The problem is that … I would not know what to say.” – *Bongi (60-64yrs, caregiver of two children).*

Some participants felt that, despite the fact that today’s generation is far better informed than they were, knowing, for example, that babies come from the “stomach”, and that the topic of sex does get covered at school, sex was still a very difficult topic for them to discuss. Reasons for this included cultural taboos, lack of SRH communication experiences in their own adolescence and general lack of information.

“Now you must discuss issues [that] weren’t discussed with you and therefore aren’t open about is a bit difficult … When the time to discuss it all of these topics … you feel overwhelmed and somewhat defeated.” – *Lerato (55-59yrs, caregiver of two children)*

#### Past experiences of talking about sex, sexuality, HIV and AIDS

A number of participants mentioned feelings of fear and experiences of corporal punishment at the hands of their parents linked to their own sexual experiences growing up. Florence described the way her parents used fear and threats when speaking about sex when she was growing up. She was also told falsehoods about where babies came from: “in suitcases delivered by nurses”. Shame was also mention as a reason to not talk about SRH:

“We are still ashamed to discuss certain issues. Sex this and that … It is difficult finding ways to tackling these issues as we never had such conversations nor was it ever taught to us at school.” – *Sindi (55-59yrs, caregiver of four children)*

#### Fear of talking about sex, sexuality, HIV and AIDS

Another source of anxiety raised by one *gogo* was based on the belief that they had grown up with that talking about sex sexuality, HIV and AIDS would encourage sexual activity.

“You see in our times, our parents felt if you taught a child about sex you were promoting the idea.” – *Sindi (55-59yrs, caregiver of four children)*

Sindi recounted that although she had spoken to her own children about sex in the past, she voiced her frustration at the fact that her children had had unplanned pregnancies in spite of the fact that she had spoken to them. She now regarded these past conversations about SRH as unsuccessful as it “failed us, because now we have these children [grandchildren] in the yard”. She expressed disappointment that her children had had babies in spite of her attempts to prevent this by speaking to them and “warning” them.

### Contextual and structural barriers to talking about sex, sexuality and HIV and AIDS encountered by *gogos*

Contextual and structural barriers played a significant role in how effective *gogos* were in their communication with their grandchildren about sex and HIV and AIDS. Although contextual and structural, these barriers were informed and defined by the personal experiences of the participants and through their perceptions of culture, gender, age and the generation gap.

#### Cultural barriers

A number of *gogos* brought up cultural issues as a barrier to talking about sex, sexuality and HIV and AIDS with their grandchildren. Communication about sex was not something participants had experienced during their own adolescence, due to the belief that parents discussing sex with their children would encourage or enable sexual activity and the fact that HIV and AIDS did not exist in their youth.

Although Sindi explained “in our culture, we are not used to that” and “it was not easy when it came to my children”, she felt very strongly that cultural beliefs restricting the sharing of open and factual information on SRH needed to be rejected and that *gogos* needed to move beyond cultural norms and address these issues with their grandchildren in order to save lives.

“There isn’t a culture that I’m committed to, to the point where I would say, I don’t do this, I don’t do that, because of my culture. Knowledge I do seek because culture doesn’t work anymore … It doesn’t work, death comes.” – *Sindi (55-59 yrs, caregiver of four children).*

#### Gender

Participants raised the issue of gender norms when speaking to grandchildren about sex, sexuality and HIV and AIDS. Not only did these gender norms apply to the *gogos* and who they spoke to, they also related to the *gogos* understanding of adolescent sexuality. Sindi explained how “[they] are used to boy children being spoken to by a man”, while one participant described how, when she tried to bring up the subject, her grandson got “uncomfortable” and tried to close down the conversation as soon as possible. In addition, these *gogos* felt that their already limited knowledge was particularly lacking when it came to speaking to their grandsons about issues pertaining to males. Sindi claimed boys would “puzzle” her, as she had no experience with them: “honestly I don’t know what I would say to a boy,” she said. Boys were advised to go for HIV testing which was a strategy for *gogos* to facilitate them getting information and advice from a trusted source.

“It is important to check, as you [a boy in her care] may not always know what the girl gets up to … so you should go and check [get tested] frequently.” *– Bongi (60-65ys, caregiver of two children).*

When talking to girls, *gogos* frequently counselled them to avoid boys as much as possible in order to protect themselves. *Gogos* regarded boys as being a threat to girls. The majority of *gogos* perceived boys as having only one agenda with regards to girls and that was to have sex. Sex with boys was communicated to girls as something that would result in sexually transmitted infections and pregnancy.

“I need you to take good care of yourself and keep clear of boys when you play. When you play, play with girls.” – *Thoko (60-64yrs, caregiver of one child).*

### Generation barrier

Several *gogos* spoke about the generation gap between themselves and their grandchildren and the need for increased understanding between them.

“There is a gap between us that needs to be bridged. They need to learn how to speak to us so we can understand one another.” – *Patience (55-59yrs, caregiver of three children)*

Respect for the older generation, and the fear of this generation losing respect, were themes that emerged during the interviews. Agnes explained that she feared she would lose the respect of her grandchildren if she spoke to them about sex, sexuality and HIV and AIDS and, having lost respect for her, her grandchildren would be more likely to participate in sexual activity.

“If I speak to them about such issues … they will end up taking you for granted. Isn't it ‘Gogo can speak to me about this so … ’ they’ll overstep their boundary” – *Agnes (55-59yrs, caregiver of three children)*

Sindi suspected some of her grandchildren’s responses, which included laughter, could have been due to the fact she was “*gogo*” and that they were possibly embarrassed by this: “Since it came with “*gogo”,* they would laugh when they see some of the things.” Sindi also mentioned that the issue of age and the generation gap as a possible reason for difficulty talking about sex, sexuality and HIV and AIDS with her grandchildren. She explained that her grandchild was not as close to her in age as he would have been to his mother.

“That is going to be really difficult, especially when they are not your [biological child]. There is no way of going beyond the fact that I’m not his mother.” – *Sindi (55-59yrs, caregiver of four children)*

Sindi and Lerato both spoke about the difficulties of trying to get their grandchildren to not only listen to their advice but also to subsequently follow this advice, rather than being influenced by other factors such as the behaviour of their peers.

“For me, it is not difficult to talk to my kids, but our kids do not want to listen. You can tell him or her something here in the house but when he is going outside to the friends, they teach their way of living.” – *Sindi (55-59yrs, caregiver of four children)*

## Discussion

All *gogo*s in the study provided care and parenting to the children they looked after and expressed a common desire to keep them safe and healthy. Despite acknowledging the need to speak to children aged 10–18 years in their care about sex, sexuality and HIV and AIDS, the experiences of the *gogo*s in doing so were extremely limited and fraught with challenges and fears. The difficulties were exacerbated by contextual and structural barriers such as culture, gender and generational issues, as well as personal challenges which included past experiences of SRH conversations and a lack of skills and knowledge around SRH**.** As a result of these difficulties most of the *gogos* had never had SRH conversations with the 10–18 year old children in their care.

Culturally, SRH communication in Africa is difficult, as many communities regard conversations around puberty, sex and sexuality as taboo [5]. Traditional values and cultural norms were seen by the *gogos* as barriers to open conversations about sex, particularly as many *gogos* had very limited experiences of these types of conversations while growing up. When, or if these conversations did occur, they were impacted by normative beliefs and the culture of their childhood and often contained misinformation. All the *gogos* spoke about the complicated and difficult SRH conversations that they had had both with their own parents and while at school. Together with past experiences of SRH conversations, gender roles and norms and generation issues appeared to impact *gogos*’ perceptions of how to communicate about SRH translating into challenges and barriers in SRH communication between the *gogos* and the third-generation children they were raising.

Research on parent–child SRH communications has found that the genders of both the parent and the child appear to play a dominant role for both parties as to the manner in which SRH discussions occurred, as women tend to speak to girls and men tend to speak to boys [5, 8]. The *gogos* in this study did not express preferences related to which gender child they spoke to, but they did comment on the fact that it was the social norm for men to speak to boys and for women to speak to girls, and they found it more difficult to speak to boys. In addition, the content of the conversations was different when speaking to boys and girls. Messages to girls often contained warnings to keep away from boys and to control their sexuality which ties in to broader gender norms about how girls should behave.

Several of the participants commented on the fact that they were *gogos* and not the mothers of these grandchildren. Generational differences presented their own specific challenges to grandparents who did not feel prepared to deal with SRH conversations [15]. As reported in a separate study by Nyasani et al. [13], the *gogos* experienced aspects of intergenerational conflict and problems with child discipline and disharmony. These added to the problems and barriers around SRH communication between *gogos* and their grandchildren. *Gogos* experienced perceived dismissal from grandchildren, feared losing respect in the eyes of their grandchildren and being laughed at by their grandchildren when attempting to initiate SRH communication.

In addition to the cultural and social obstacles discussed above, the *gogos* explanation for the lack of SRH communication in their youth was that their parents feared that discussion and knowledge of sexual topics would lead to children engaging in sexual activities. In addition, the conversations about sex that the *gogos* had had with their own parents had often been negative and punitive. Parental involvement in sexual communication is generally a new notion for South African families [23] and many of the *gogos* in the parental role found themselves having the same types of negative conversations about SRH when speaking to grandchildren about sex, sexuality and HIV and AIDS as they had had with their own children.

The fear that speaking to children about sexual behaviour would promote sexual activity is common theme in sub-Saharan Africa [5]. The *gogos* explained that their parents shared this fear and it was at least partly responsible for the lack of perceived efficacy on the part of the *gogos* in addressing SRH with their own children. Many *gogos* reported limited skills or experience in how to have conversations about sex, sexuality and HIV. A study by Cornelius et al. [18] found that African American grandmothers did not fear that talking about sex would promote sexual behaviour in their grandchildren, and embraced the idea of talking to their grandchildren about sex [18]. The results of our study aligns with Cornelius et al. [18] as fear of promoting sexual activity was not mentioned by the *gogos* as a barrier to having SRH conversations their grandchildren or children in their care. The *gogos* in this study showed that there has been a shift away from this belief based on their lived experiences that not talking sex, sexuality and HIV does not prevent sexual behaviour or HIV infection. In fact, many of them felt that SRH conversations were necessary.

Personal beliefs and experiences cannot be totally discounted as a barrier to SRH discussions, as many parents are from a generation where discussing sexuality topics was linked to embarrassment [5, 8]. Although grandparents can be receptive to discussing sex and sexuality with their grandchildren, they experience different levels of comfort with these conversations [18]. In our study *gogos* expressed discomfort and a lack of confidence and ability to acknowledge, confront or discuss the difficult and uncomfortable topic and issues of adolescent sexual behavior and felt they lacked the skills to do this although this could potentially be mitigated by providing support for the *gogos,* enabling them to conduct the discussions more effectively.

Despite increasing evidence of programmes targeting grandmothers to support SRH communication having positive outcomes [20, 21], only one *gogo* in our study had received any prior HIV training. A South African study in the Eastern Cape reported that grandparents were unsuccessful in communicating SRH matters to youth and concluded that grandparents should be supported in SRH conversations with their grandchildren [25]. A similar need was identified through our research, with the participants acknowledging the value of these conversations in reducing HIV infection and unintended pregnancy. Our findings clearly demonstrate the need to engage with *gogos* to increase effective SRH communications as *gogos* stated that SRH conversations would be easier if they, and the children in their care, had access to workshops which built communication skills on these topics.

A number of studies in South Africa have commented on the importance of the role of extended family and caregivers other than parents [23, 24, 26–28] and this study reinforces the view that it is essential to examine the changes in family composition in SRH conversations in South Africa [23, 26–28]. In addition, this study supported findings in other research [17, 22] for the need for healthcare providers and programmes to recognise that there are differences in SRH communication and conversations between parents and grandparents. Aubel (2005) showed that the concern that grandmothers might be biased and unable to adapt to new ideas is incorrect and that this should not be a reason that interventions do not make use of older people as drivers of community and family-based interventions [29]. Grandmothers are in fact open and receptive to change and new ideas and can be very powerful resources in promoting health changes [29]. Like Aubel (2005), our findings challenged the stereotype of grandmothers as being conservative and resistant to change as all the *gogos* expressed a desire to be able to have informed and effective SRH conversations with the grandchildren they were looking after or raising.

The findings need to be considered in the light of the following limitations related to the collection of data and the role of the lead researcher. She had prior links to Ratang Bana which could have influenced her interpretation of the findings and the data analysis because she was aware of the difficulties experienced by the participants. In addition, the researcher was sympathetic to parenting issues as a mother of adolescent children herself and had established relationships with some of the participants prior to the commencement of the research. Furthermore, although the participants were typical of *gogos* in Alexandra, they were all accessing support at a small, local NGO which could have influenced their experiences and how they engaged in the interviews. In addition, only a small number [10] of older women met the criteria to participate in this study due to the NGO being a small community organisation. Saturation was reached on several topics vindicating the decision to go with a relatively small number of study participants. However, some of these limitations were mitigated by having an experienced interviewer who did not have any prior connection with the organisation, spoke the same languages and was a similar age to participants.

Another limitation in this study is that the ages of the children being referred to in the *gogos*’ interviews was not recorded and it is indeed possible that SRH conversations were easier with older children (15–17 years of age) than younger children (10–14 years). Furthermore, it is not possible in some instances to know if the *gogos* were referring to boys or girls in their comments. It is likely that the experiences of SRH communication for *gogos* differ across ages and genders so future research should look more carefully at this.

## Conclusion

Talking about sex is not easy for *gogos* due to personal and contextual and structural barriers. Personal barriers for the *gogo* included the difficulty and fear of talking about SRH and their own experiences growing up of not talking about sex, sexuality and HIV and AIDS. Contextual and structural barriers to talking about sex, sexuality and HIV and AIDS included cultural beliefs and practices, gender issues and the generation gap experienced between the *gogos* and the children they were raising. Many grandmothers and older female members in the community, the *gogos*, are primary caregivers of children and are fulfilling parental roles in raising children. This parental role needs to be recognised and issues affecting *gogos* need to be acknowledged. *Gogos* need to become part of, if not particularly targeted by, interventions to improve the SRH outcomes of the children they are raising.

## Supplementary Information

**Additional file 1.** Communication between grandmothers and their grandchildren. Interview Guide 1 (for in depth interview before skills training workshop).

## Data Availability

Data and material can be requested from the corresponding author.

## Abbreviations

AIDS: Acquired Immune Deficiency Syndrome
HIV: Human Immuno-Deficiency Virus
NC: Nicola Christofides
SHR: Sexual Reproductive Health
