# Supplementary material for: “Knowledge I seek because culture doesn’t work anymore … It doesn’t work, death comes”: the experiences of third-generation female caregivers (gogos) in South Africa discussing sex, sexuality and HIV and AIDS with children in their care
Source: BMC Public Health. 2021 Mar 9;21:470. doi: 10.1186/s12889-021-10494-5 (PMC7941880; doi:10.1186/s12889-021-10494-5)
Supplement: Supplementary file 1 — Additional file 1. Communication between grandmothers and their grandchildren. Interview Guide 1 (for in depth interview before skills training workshop). [file 12889_2021_10494_MOESM1_ESM.docx]

## Additional Files

## *Additional File 1*

## *Communication between grandmothers and their grandchildren*

*Interview Guide 1 (for in depth interview before skills training workshop)*

Good day and welcome. My name is Thuli. Thank you for giving me your time. I am researching communication between grandmothers and their teenage grandchildren on sex, sexuality and HIV and AIDS to find out your feelings and experiences of talking to your adolescent grandchildren about sex, sexuality and HIV and AIDS.

1)Tell me about your grandchildren and your relationship with them

Probes

- activities- things they do

- needs- demands/requirements from grandchildren on grandmothers

- problems- school attendance, respect, and disobedience

2) How do you feel about talking to your grandchildren about sex and HIV and AIDS

Probes

What could make it easier to talk to your grandchildren, if anything

What are the difficulties, if any

Who should talk to children about sex and HIV and AIDS

3) Tell me about the last time you spoke to your grandchildren about sex and HIV and AIDS, if at all

4) If you have you never spoken to them- tell me the reasons
